# Progressive dementia associated with ataxia or obesity in patients with *Tropheryma whipplei *encephalitis

**DOI:** 10.1186/1471-2334-11-171

**Published:** 2011-06-15

**Authors:** Florence Fenollar, François Nicoli, Claire Paquet, Hubert Lepidi, Patrick Cozzone, Jean-Christophe Antoine, Jean Pouget, Didier Raoult

**Affiliations:** 1 Unité des rickettsies, URMITE CNRS-IRD UMR 6236, IFR 48, Faculté de médecine, Université de la Méditerranée, Marseille, France; 2 Service de Neurologie, Hôpital de La Timone, Marseille, France; 3 Centre de Résonance Magnétique Biologique et Médicale and Centre d'Exploration Métabolique par Résonance Magnétique, UMR CNRS 6612, Faculté de Médecine, Université de la Méditerranée, and Hôpital de La Timone, Marseille, France; 4 Memory Center, Department of Pathology, Lariboisière Fernand Widal Hospital University of Paris 7 and INSERM UMRS 839, France; 5 Service de Neurologie, CHU de Saint-Etienne, France

## Abstract

**Background:**

*Tropheryma whipplei*, the agent of Whipple's disease, causes localised infections in the absence of histological digestive involvement. Our objective is to describe *T. whipplei *encephalitis.

**Methods:**

We first diagnosed a patient presenting dementia and obesity whose brain biopsy and cerebrospinal fluid specimens contained *T. whipplei *DNA and who responded dramatically to antibiotic treatment. We subsequently tested cerebrospinal fluid specimens and brain biopsies sent to our laboratory using *T. whipplei *PCR assays. PAS-staining and *T. whipplei *immunohistochemistry were also performed on brain biopsies. Analysis was conducted for 824 cerebrospinal fluid specimens and 16 brain biopsies.

**Results:**

We diagnosed seven patients with *T. whipplei *encephalitis who demonstrated no digestive involvement. Detailed clinical histories were available for 5 of them. Regular PCR that targeted a monocopy sequence, PAS-staining and immunohistochemistry were negative; however, several highly sensitive and specific PCR assays targeting a repeated sequence were positive. Cognitive impairments and ataxia were the most common neurologic manifestations. Weight gain was paradoxically observed for 2 patients. The patients' responses to the antibiotic treatment were dramatic and included weight loss in the obese patients.

**Conclusions:**

We describe a new clinical condition in patients with dementia and obesity or ataxia linked to *T. whipplei *that may be cured with antibiotics.

## Background

Whipple's disease is a paradigm of the evolution of infectious disease knowledge [1]. The disease was first described in 1907 by Whipple [2], who based it on anatomopathological lesions identified in a patient at autopsy. For many years, it was considered to be a metabolic disorder; however, in 1952, a bacterial origin became suspected when antibiotic treatment proved effective [3]. The first molecular identification of the bacterium associated with Whipple's disease (*Tropheryma whipplei*), as well as the first culture, created a new field [4,5]. *T. whipplei *has been identified in the saliva and stool specimens of healthy people [1]. The well-known and classic form of Whipple's disease, which is characterised by periodic acid-Schiff (PAS)-stained bacilli in infected small-bowel macrophages, represents only one rare clinical form of the infection that can be caused by *T. whipplei*. Indeed, the bacterium has also been involved in subacute or chronic infections without gut lesions, such as endocarditis, encephalitis, uveitis, adenopathy and pulmonary and osteoarticular infections [1,6-9]. The diagnosis and management are different for each of these infections [1,9,10]. Finally, acute *T. whipplei *infections, such as pneumonia [11,12], gastroenteritis [13] and transit bacteraemia [14], have recently been reported.

Neurologic forms are often the most serious manifestations of *T. whipplei *infection, particularly with respect to relapse treatment failure [15]. It has been noticed that relapses of classic Whipple's disease can be exclusively cerebral, without any peripheral manifestations [1]. We recently diagnosed infection with *T. whipplei *in a patient who presented with progressive dementia and recent-onset obesity and responded dramatically to antibiotic treatment. This observation encouraged us to document additional cases of *T. whipplei *infections using brain biopsies and cerebrospinal fluid specimens.

## Methods

### Patients

#### Patients from the study

From January 2001 to June 2009, 824 cerebrospinal fluid specimens and 16 brain biopsies from patients without a previous diagnosis of Whipple's disease were analysed using PCR for *T. whipplei *in our laboratory in Marseille, France, which is a reference centre for *T. whipplei *diagnosis in our country. All samples were collected as part of routine clinical management, and they were sent to our laboratory for the detection of all microorganisms potentially responsible for encephalitis. Our diagnostic criteria for *T. whipplei *encephalitis required at least two positive PCR assays targeting 2 different sequences on 2 different cerebrospinal fluid specimens, performed as previously reported, or positive PCR assays on brain biopsies [16,17] and negative results PAS staining of gastric and small-bowel specimens, plus an absence of meningitis, myelitis, and other organ involvement. Our study is in compliance with the Helsinki declaration. The local ethics committee from IFR 48 (Marseille, France) approved this study. Written informed consent was obtained from the patient for the use of information in this case report and any accompanying images.

#### Patients from the literature

A MEDLINE (National Library of Medicine, Bethesda, MD) search of the literature from 1966 to April 2010 was performed. The following terms were used, both alone and combined: *Tropheryma whipplei*, *Tropheryma whippelii*, Whipple's disease, brain, cerebral, encephalitis and cerebrospinal fluid. References prior to 1966 were searched by cross referencing. Our initial analysis indicated considerable confusion in the literature regarding the specific diagnosis of *T. whipplei *encephalitis. We then applied rigorous criteria to classify the diagnosis as certain, possible or excluded. The diagnosis was considered certain only for patients with positive *T. whipplei *PCR, which allows the specific identification of the bacterium, and those with a negative PAS staining from gastric and small-bowel biopsies. Patients for whom digestive biopsies were not available were excluded from further analysis. Patients for whom the diagnosis was based solely on PAS staining of brain biopsies were also excluded; among 12 brain biopsies sent to our laboratory to confirm *T. whipplei *encephalitis (Table 1), all showed a nonspecific inflammatory process of the brain with numerous PAS-positive macrophages in the white matter, but specific immunohistochemistry yielded negative results. Patients who exhibited structures suggestive of bacterium under electron microscopy, along with positive PAS staining on brain biopsies, were classified as possible *T. whipplei *infection cases, as were patients with a diagnosis based exclusively on oculomasticatory myorhythmia. This rigorous strategy resulted in fewer patients with localised neurologic manifestations than have previously been reported [1].

**Table 1:** Summary of 14 brain biopsies from patients with encephalitis analysed in our laboratory using PAS staining, immunohistochemistry with polyclonal rabbit antibodies specifically directed against *T. whipplei *and specific PCR

			Brain biopsies		
**Patients**	**Sex/Age**	**Country of origin**	**PAS staining**	***T. whipplei *immunohistochemistry**	***T. whipplei *PCR**

**False-positive PAS staining associated with negative immunohistochemistry**					

***Excluded diagnosis of T. whipplei encephalitis***					

*1	F/67	Ireland	Positive	Negative	Negative
*2	M/25	Canada	Positive	Negative	Negative
*3	M/42	France	Positive	Negative	Negative
*4	M/37	Belgium	Positive	Negative	Negative
*5	NA/NA	Japan	Positive	Negative	Negative
*6	M/34	France	Positive	Negative	Negative
*7	M/64	France	Positive	Negative	Negative
*8	M/66	France	Positive	Negative	Negative

***Diagnosis of T. whipplei encephalitis not available due to lack of PCR ***^***2***^					

*9	M/73	Japan	Positive	Negative	NA ^1^
*10	M/14	USA	Positive	Negative	NA ^1^
*11	NA/NA	South-Africa	Positive	Negative	NA ^1^
*12	M/NA	Canada	Positive	Negative	NA ^1^

**False-negative PAS staining and immunohistochemistry**					

*^2^Patient 1 in this study	M/39	France	Negative	Negative	Positive
*^2^Patient 5 in this study	F/33	France	Negative	Negative	Positive

NA: Not available

*: All samples from these patients were analysed in our facilities.

^1^: PCR assays were not available for these patients because no fresh specimens were sampled and the quality of the DNA extracted from the paraffin-embedded specimens was poor, not allowing a conclusive diagnosis.

^2^: Patients were seen and followed by one of the authors (DR) in consultation.

## Methods

### PCR assays, serologic studies and isolation procedures

PCR assays were performed as previously reported [16-20]. Two hundred μl cerebrospinal fluid specimens were submitted for DNA extraction using the QIAamp DNA MiniKit (Qiagen, Hilden, German) according to the manufacturer's recommendations. PCR mixes were prepared using a Fast-Start DNA Master SYBR Green kit (Roche, Mannheim, Germany) following the manufacturer's instructions. Quantitative PCR (qPCR) was performed in a LightCycler thermocycler (Roche biochemicals, Mannheim, Germany). For each assay, positive and negative controls were used. At intervals of 5 samples, negative controls (water, PCR mix and human samples) were evaluated. A tenfold dilution of a standard suspension of 10^6 ^*T. whipplei *strain Marseille-Twist was used as a positive control and for quantification, as previously reported [21]. The quality of DNA extraction from samples was estimated by PCR targeting a housekeeping gene coding β-actin. If a first PCR assay was positive, the result was systematically confirmed by a second PCR assay using a second set of primer pairs. In the event of discrepancies between the two PCR assays or incorrect controls, samples were submitted to new DNA extraction and/or new qPCR assays.

The PCR primers that we used have evolved with improved knowledge about *T. whipplei*. Between January 2001 and February 2004, all samples were tested using our regular qPCR, targeting a 489-bp fragment of the 16S-23S rRNA gene intergenic spacer using the primers tws3f (5'-CCGGTGACTTAACCTTTTTGGAGA) and tws4r (5'-TCCCGAGGCTTATCGCAGATTG), as previously reported [17]. If this assay was positive, a 650-bp fragment of the *rpoB *gene using the primers TWRPOB.F (5'-TTTTTCCGGCGTGCGCTCAA) and TWRPOB.R (5'-TTTCTCCGAGGTTGCGGTGC) was performed [17]. For all of the assays, when an amplified product was detected, *T. whipplei *was systematically confirmed by sequencing, as previously reported [19]. Since October 2003, the availability of the *T. whipplei *genome has offered the option of using repeated sequences of *T. whipplei *for highly sensitive and specific PCR assays [16]. Subsequently, a PCR targeting repeated sequences of *T. whipplei *was developed that, when an amplified product was detected, confirmed the identification of *T. whipplei *by sequencing (between October 2003 and March 2004) or by using specific oligonucleotide Taqman* probes (since April 2004). From October 2003 to March 2004, *T. whipplei *qPCR targeting a 164-bp sequence of the bacterium incorporated the primer pairs 53.3F (5-AGAGAGATGGGGTGCAGGAC) and 53.3R (5'-AGCCTTTGCCAGACAGACAC) into the reaction mix. If this assay was positive, the result was confirmed by a second assay using a second set of primer pairs, 342F (5'-AGATGATGGATCTGCTTTCTTATCTG) and 492R (5'-AACCCTGTCCTGCACCCC), which targeted a different DNA sequence. Since April 2004, the reaction mix has included *T. whipplei *qPCR targeting a 155-bp sequence of the bacterium and incorporating the primer pair TW27F (5'-TGTTTTGTACTGCTTGTAACAGGATCT) and TW182R (5'-TCCTGCTCTATCCCTCCTATCATC) and a Taqman* probe (27F-182R, 6-FAM-AGAGATACATTTGTGTTAGTTGTTACA-TAMRA). If this assay was positive, the result was confirmed by a second assay using a second set of primer pairs TW13F (5'-TGAGTGATGGTAGTCTGAGAGATATGT) and TW163R (5'-TCCATAACAAAGACAACAACCAATC) and a Taqman* probe (13F-163R, 6-FAM-AGAAGAAGATGTTACGGGTTG-TAMRA) targeting a different 150-bp sequence [20-22]. Finally, all the cerebrospinal fluid specimens prior to October 2003 were retrospectively tested using PCR targeting repeated sequences, and the specimens sampled between October 2003 and February 2004 were tested using both regular and repeat PCR.

Serologic studies were performed as previously reported [23]. Cerebrospinal fluid specimens were cultivated using both cell-culture and cell-free culture media, as previously described [24,25].

### Brain magnetic resonance imaging and spectroscopy protocols

Brain magnetic resonance examinations were performed on a 1.5 T Magnetom Vision Plus system (Siemens, Erlangen, Germany). The imaging protocol included one axial FLAIR sequence (TR = 8000 ms, TI = 180 ms, TE = 110 ms) and one axial T1-weighted sequence (TR = 644, ms TE = 15 ms) performed before and after contrast agent administration (0.2 ml/kg of Gadolinium-DTPA, Guerbet, Paris, France). Magnetisation transfer imaging was performed using two axial proton-density weighted FLASH sequences (TR = 500 ms, TE = 4.7 ms, 21 slices, thickness = 5 mm, flip angle = 30°, without and with MT saturation: 1.5 kHz off resonance, 500°, FOV = 240 mm, matrix size = 256 × 256). Diffusion-weighted imaging was performed using a single-shot EPI sequence (b = 0, 500, 1000 s/mm^2^) applied in the x, y and z directions (19 slices, thickness = 5 mm, matrix size = 128 × 128, FOV = 256 × 256 mm^2^). Apparent diffusion coefficient (ADC) maps were reconstructed using this sequence. Single voxel proton magnetic resonance spectroscopy (SVS) included STEAM acquisition at an echo time of 20 ms. TR was 1500 ms. One voxel was centred on the medulla oblongata lesion, while the reference voxel was located on a contiguous area (the pons) that appeared normal on all MR sequences. The voxel volume was adapted to the size of the lesion. Several normal brain metabolites are detected using the STEAM 20 ms sequence: N-acetylaspartate (NAA) is an amino acid that is present almost exclusively in neurons; reduced levels may correspond to neuronal death or injury. Myoinositol (MI) is a sugar that is only present in glia. It reduces when these cells experience specific damage and increases in cases of glial activation or proliferation (in glial tumours or reactive gliosis) [26]. Other molecules are detected under pathological conditions. Lactate and lipids or macromolecules may be detected when there is a local ischemia that eventually leads to necrosis (as with ischemic stroke and some tumours). Lactate may also be detected in relation to a macrophage infiltration. High lactate and lipid levels are also detected in cases of abscess. Other molecules such as succinate, acetate, alanine and other amino acids can be detected using *in vivo *magnetic resonance spectroscopy in cases of brain abscess, but only when the infectious lesion is cystic. Creatine/phosphocreatine (tCr) is a ubiquitous metabolite involved in energy storage. It is a marker of overall cellular density. Choline (Cho) is mostly involved in cell membrane metabolism, and it is thus a membrane turnover marker. It increases whenever there is cellular proliferation (as with brain tumours), inflammation or membrane catabolism (as with multiple sclerosis).

### Statistical analysis

Data were analysed using EpiInfo software, Version 3.4.1 (Centers for Disease Control and Prevention, Atlanta, GA, USA). *P *< 0.05 was considered statistically significant.

## Results

### Index patient

**Figure 1:**
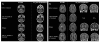
**Figure 1a and 1b. Magnetic resonance examinations of Patient 1**. MR examinations were performed on a 1.5 T Magnetom Vision Plus system (Siemens, Erlangen, Germany). The imaging protocol included one axial and coronal T2-weighted sequence and one axial T1-weighted sequence, performed before and after contrast agent administration not at diagnosis but before treatment, after 18 months of treatment, at relapse, and after 17 months of a new treatment. A multimodal MRI of Patient 1 at the time of the diagnosis disclosed multiple brain lesions located in the medulla oblongata, the hypothalamus, the internal side of the right temporal lobe, the bilateral fornix, the lenticular nuclei and the head of caudate nuclei. These nonspecific, but unusual, lesions have a high signal intensity on FLAIR images and low signal intensity on MT images. They are slightly enhanced on T1-weighted images after gadolinium administration. Complete normalisation of the FLAIR abnormalities after 18 months of treatment were observed. A new lesion appeared in the left medulla oblongata during the relapse; it was enhanced after gadolinium injection. FLAIR abnormalities reappeared during the relapse, and lenticulocaudate gadolinium enhancement was also observed. A complete disappearance of the lesions after 17 months of treatment and the complete disappearance of gadolinium enhancement were also observed, in accordance with clinical improvement.

A 39-year-old male (Patient 1) was hospitalised for cognitive impairment and choreiform movements. His medical history included a nearly 3-week-long period of unexplained gastroenteritis 4 years earlier. Over a 3-year period, the patient developed hepatitis and obesity (a 25-kilogram weight gain) associated with hypercholesterolemia, hypertriglyceridemia and Type II diabetes. Hormonal evaluation showed peripheral hypoandrogenia with elevated levels of follicle-stimulating hormone. Brain magnetic resonance imaging (MRI) did not show any abnormalities. One year prior to hospitalisation, he developed dementia, dysarthria, and apathy, followed by involuntary and almost permanent choreiform movements of the left hemibody and abnormal buccofacial movements. No blood cell count abnormalities or C-reactive protein increases were observed. An increase in hepatic transaminases was identified. A lumbar puncture showed a high protein level (4 g/l) with a normal glucose level. Examinations with brain computer tomography (CT) and brain MRI and spectroscopy were abnormal (Figure 1 and additional file 1, Figure S1). The MRI disclosed multiple unusual lesions corresponding to multiple brain lesions located in the medulla oblongata, hypothalamus, internal side of the right temporal lobe, fornix, lenticular nuclei and head of the caudate nuclei. These lesions showed high signal intensity on FLAIR images and low signal intensity on MT images, and they were slightly enhanced on T1-weighted images after gadolinium administration. Spectroscopy was characterised by a decrease in the NAA and MI peaks (consistent with neuronal and glial injury) and an increase in lipids and macromolecules, whereas lactate and other amino acids suggestive of an abscess were not detected. Finally, the absence of an increase in the choline-creatine ratio ruled out a demyelinating lesion or neoplasm. A brain biopsy was taken from the right striatum; it revealed astrocytic gliosis and vascular proliferation. No granulomas were observed. PAS staining and *T. whipplei *immunohistochemistry were negative. Regular PCR targeting the intertransgenic spacer (ITS) was negative for this brain biopsy; however, repeat PCR was positive for both the biopsy specimen and cerebrospinal fluids. One month after beginning specific treatment (doxycycline 200 mg, hydroxychloroquine 200 mg 3 times and trimethoprim-sulfamethoxazole 320 + 1,600 mg 3 times each per day), significant improvements were observed; however, cerebrospinal fluid showed persistent high protein levels, and repeat-PCR was still positive. After 2 months, the clinical examination was normal and, surprisingly, the patient had lost 17 kg. One year after the start of the treatment, the patient's obesity had completely regressed, his hepatic tests were normal and he presented no evidence of diabetes; furthermore, brain MRI revealed that the majority of the lesions had disappeared. After 18 months of treatment, brain MRI and spectroscopy results were normal. The repeat PCR for cerebrospinal fluids was negative. The treatment was stopped. Fourteen months later, he experienced a clinical relapse, with weight gain (10 kilograms), the reappearance of diabetes, abnormal choreic movements, headache, difficulty concentrating and increased hepatic transaminases. Brain MRI and spectrometry showed signs of relapse, but *T. whipplei *repeat PCR for CSF was negative. A treatment based on doxycycline, hydroxychloroquine and sulfadiazine (1,500 mg four times per day) was started. All clinical abnormalities resolved, including obesity, similar to the patient's response to the first treatment. After 17 months of treatment, the patient's brain MRI was normal and spectroscopy was clearly improved. After 52 months, the treatment was stopped. Seventeen months after the completion of treatment, the patient showed no sequelae and felt well.

### Other patients

The characteristics of 4 other patients with *T. whipplei *encephalitis (Patients 2, 3 and 4), seen by one of the authors (DR) in consultation, and 15 patients from the literature (8 with confirmed *T. whipplei *encephalitis and 7 with a possible diagnosis) are summarised in an additional file 2, Table S1 [27-43]. Seventy-four patients with Whipple's disease with neurologic manifestations and sufficient clinical information were also identified from the literature review [15,27,44-54]. Comparisons among the different groups are summarised in Tables 2 and 3.

**Table 2:** Features of patients with Whipple's disease [1], patients with Whipple's disease with neurological manifestations and patients with *T. whipplei *chronic encephalitis

Feature	Patients with Whipple's disease	Patients with Whipple's disease and neurological manifestations	Patients with certain *T. whipplei *encephalitis	Patients with possible *T. whipplei *encephalitis	Patients with certain or possible *T. whipplei *encephalitis
	**No./total no. (%)**				

Male sex	770/886 (87)	57/74 (77)	8/13 (61.5)	4/7 (57)	12/20 (60)
Age	NA	50 ± 13	50 ± 13	52 ± 12	51 ± 12
Arthralgia or arthritis	244/335 (73)	49/74 (66)	1/11 (9)	3/7 (43)	4/18 (22)
Chronic diarrhoea	272/335 (81)	30/74 (40.5)	0/12	0/6	0/18
Weight loss	223/240 (93)	35/57 (47)	1/11 (9)	0/6	1/17 (6)

NA: Not available

**Table 3:** Clinical features* of 74 patients with Whipple's disease (*i.e.*, patients with histological digestive involvement characterised by positive PAS staining) with neurologic manifestations and those with certain (13) and possible (7) *T. whipplei *chronic encephalitis without digestive lesions

Neurologic manifestations	Whipple's disease with neurologic manifestations	Certain *T. whipplei *encephalitis	Possible *T. whipplei *encephalitis	Certain and possible *T. whipplei *encephalitis
Number of patients	74	13	7	20

Cognitive impairment	53 (72%)	9 (69%)	7 (100%)	16 (80%)
**Ataxia**	12 (16%)	**9 (69%; *p *< 10**^**-3**^**)**	**4 (57%; *p *= 0.02)**	**13 (65%; *p *< 10**^**-3**^**)**
Supranuclear ophthalmoplegia	29 (39%)	5 (38%)	4 (57%)	9 (45%)
Hypothalamic manifestations	24 (32%)	4 (31%)	2 (29%)	6 (30%)
**Dysarthria**	9 (12%)	4 (31%)	3 (43%)	**7 (35%; *p *= 0.03)**
**Cerebellar forms**	4 (5.5%)	**3 (23%; *p *= 0.06)**	**3 (43%; *p *= 0.03)**	**6 (26%; *p *= 0.005)**
**Myorhythmia**	10 (13.5%)	4 (31%)	3 (43%)	**7 (35%; *p *= 0.045)**
Oculomasticatory myorhythmia	7 (5.5%)	2 (17%)	2 (29%)	4 (20%)
Upper motor neuron disorder	11 (15%)	4 (31%)	3 (43%)	7 (35%)
Decreasing level of consciousness	25 (35%)	2 (15%)	6 (75%; p = 0.01)	8 (40%)
Myoclonus	29 (39%)	3 (23%)	1 (14%)	4 (20%)
Depression	11 (15%)	3 (23%)	1 (14%)	3 (15%)
Personality changes	22 (30%)	2 (15%)	2 (29%)	4 (20%)
Headache	10 (13.5%)	1 (8%)	2 (29%)	3 (15%)
Apathy	15 (20%)	1 (8%)	1 (14%)	2 (10%)
Muscle weakness	14 (19%)	2 (15%)	2 (29%)	4 (20%)
Seizures	13 (18%)	3 (23%)	1 (14%)	4 (20%)
Nystagmus	11 (15%)	3 (23%)	1 (14%)	4 (20%)
Extrapyramidal movement disorder	8 (11%)	2 (17%)	2 (29%)	4 (20%)

Significant *p *values are noted in bold.

* Neurological signs were defined as follows: "Decreasing level of consciousness" included somnolence, lethargy, and coma. "Cognitive impairment" included abnormalities of orientation, memory, or reasoning. "Hypothalamic manifestations" included polydipsia, hyperphagia, sexual impotence, changes in the sleep-wake cycle and insomnia, but not isolated somnolence. Oculomasticatory myorhythmia was defined as pendular vergence oscillations of the eyes that were synchronous with masticatory myorhythmia. Both were classified as myorhythmia. "Myoclonus", which is nonrhythmic, was distinguished from myorhythmia. "Cerebellar forms" were considered when a patient presented dysarthria and ataxia.

*T. whipplei *encephalitis has two consistent findings: progressive cognitive impairment and the eventual onset of obesity or ataxia. For our 5 patients, the only diagnostic finding was a positive PCR of for cerebrospinal fluid and brain biopsies. This disease arises from the isolated involvement of *T. whipplei *localised to the central nervous system, which responded to antibiotic treatments. Among our patients and those in the literature, all 3 who did not receive antibiotics (because *T. whipplei *infection was diagnosed after post-mortem examination) died, compared with only 3 deaths among those who received antibiotics. This difference was statistically significant (*p *= 0.01). No specific brain MRI abnormalities were observed. Six out of 18 patients had a normal brain MRI.

### PCR assays, isolation procedure and serologic studies

Among the 824 cerebrospinal fluid specimens, 7 (0.85%) tested positive for *T. whipplei *DNA with PCR; 2 out of 16 brain biopsies were PCR positive, including a sample from one patient for whom cerebrospinal fluid was negative. Negative and positive controls were correct, including the amplification of the gene coding β-actin. All of our attempts to cultivate *T. whipplei *from the cerebrospinal fluid specimens of our 6 patients with *T. whipplei *encephalitis and positive PCR samples were unsuccessful; in contrast, we established 9 strains of *T. whipplei *from the cerebrospinal fluid specimens of patients with classic Whipple's disease with associated neurologic manifestations (6/8), asymptomatic neurologic involvement (2/4), or neurologic relapse (1/3) (published and unpublished data) [1]. This difference was statistically significant (*p *< 0.017). We hypothesised that this difference might be linked to a lower amount of bacteria in the cerebrospinal fluid specimens of patients with *T. whipplei *encephalitis. Serum sample serologic tests were available from 5 patients with *T. whipplei *encephalitis. Of these, 2 showed no reaction and 3 showed low-level but specific responses to the deglycosylated proteins of *T. whipplei*, as has been observed in patients with classic Whipple's disease [23].

## Discussion

We describe a clinical phenomenon of patients with *T. whipplei *who present with isolated brain involvement and respond dramatically to antibiotic treatment. Their condition was poor and worsening at the time of the diagnosis. As in the review of the literature, the antibiotics address the progressive cognitive impairment. In our index patient, antibiotic therapy not only improved his clinical status but also caused a reversal of obesity, both at the time of the initial diagnosis and upon relapse. These observations are reminiscent of those described in 1952 after the first successful use of an antibiotic (chloramphenicol) to treat Whipple's disease, which suggested that the disease had a bacterial origin [3].

The amount of *T. whipplei *DNA in the cerebrospinal fluid specimens of the patients described in our series was low, as demonstrated by our quantitative PCR assays; however, we are confident that these results are accurate, because we performed 2 PCR tests on 2 different samples to confirm the diagnosis. Our attempts to isolate a strain from the cerebrospinal fluid specimens failed, although we were able to isolate strains from other cerebrospinal fluids from patients with classic Whipple's disease with a good percentage of success (60%). Even in the 2 brain biopsies from our patients, only very low copies of *T. whipplei *DNA were detected. Moreover, these 2 brain biopsies were PAS negative and *T. whipplei *immunohistochemistry negative. It is also important to note that these data are different from those of patients in the literature with positive PAS staining of brain biopsies. Indeed, there is a link between the clinical picture of our patients and *T. whipplei*, but we were unable to identify histological findings compatible with described *T. whipplei-*associated diseases. However, it has also recently been reported that *T. whipplei *DNA is highly frequent in the skin biopsies of patients with classic Whipple's disease, whereas PAS staining is significantly less sensitive in the same group [55]. Furthermore, these bacteria are alive, as a positive culture from a skin specimen has been obtained. Overall, these data show that PAS staining, which was long considered the gold standard for diagnosing *T. whipplei *infection, is frequently negative, despite the presence of *T. whipplei *infection.

Overall, only 4 (18%) out of 18 patients reviewed here reported a history of arthralgia, despite its description as a cardinal symptom of classic Whipple's disease (73%) [1]. Our study patients reported no chronic diarrhoea, whereas it is observed in 81% of patients with classical Whipple's disease and 40.5% of patients with a neurologic presentation of Whipple's disease [1]. It is interesting to note that 3 of our 5 patients reported a history of unexplained acute diarrhoea prior to the onset of neurologic symptoms, which may correspond to primary infection with *T. whipplei*, as described previously in young children [13]. Weight loss was observed for none of our patients and only one from the literature versus 93% of patients with classic Whipple's disease and 47% of patients with a neurologic presentation of Whipple's disease [1]. Even more interesting, 2 of our 5 patients developed obesity during the primary illness or during the relapse. Several viruses have been reported to contribute to weight gain in animals [56]. In humans, one adenovirus has been also identified as a possible contributor to weight gain [56]. The most likely mechanisms are a central effect on appetite and energy expenditure associated with hypothalamic infiltration by the pathogen. This clinical picture may be a particular form caused by *T. whipplei*. It appears that this clinical entity resembles a localised cerebral relapse of classic Whipple's disease when bacterial multiplication is relatively controlled. Another interesting observation is the deficient humoural responses against *T. whipplei *in patients with *T. whipplei *encephalitis, as have been described in patients with Whipple's disease compared with asymptomatic carriers [23].

This clinical entity seems rare, as only 0.85% of cerebrospinal fluid specimens of patients with unexplained dementia or encephalitis tested in our laboratory were positive. However, our technique may lack sensitivity in some cases, as the repeat PCR assays were unable to detect *T. whipplei *when brain MRI-spectroscopy showed lesions compatible with reactive disease at the time of clinical relapse. Furthermore, the lack of information about antibiotic treatment status prior to cerebrospinal fluid sampling for most of the patients must also lead us to regard this prevalence cautiously, as antibiotic use might explain some negative PCR results. As seen in our patients, PAS staining of brain biopsy specimens with *T. whipplei *also lacks sensitivity. Furthermore, this tests lacks specificity, as perivascular aggregates of foamy PAS-positive macrophages in a reactive gliosis setting can be seen in other macrophage-rich cerebral disorders, such as demyelinating diseases, cerebral infarction, and a host of infectious diseases [57]. The results of PAS-staining of brain biopsy specimens can neither confirm nor exclude the diagnosis of *T. whipplei *encephalitis. The development of cognitive impairments associated with ataxia or obesity should point the clinician to a diagnosis of *T. whipplei *encephalitis.

PCR testing of cerebrospinal fluid specimens for *T. whipplei *in neurological patients is clinically reasonable, as the disease is fatal without specific treatment. As is the case for all diagnoses performed using PCR, caution is necessary, and a rigorous strategy should be applied when performing and interpreting the analyses due to the risk of false-positive results [58]. A carefully checked positive PCR in cerebrospinal fluid specimens is sufficient for diagnosis. It is important to remember the patient for whom a Whipple's disease diagnosis was ruled out by negative PAS staining, despite positive PCR, and then finally confirmed using both techniques at the patient's autopsy [59].

Another question concerns the management of this presentation. Advances in knowledge, with *in vitro *tests and full genome sequencing of *T. whipplei*, have shown that the usual long-term treatment based on trimethoprim-sulfamethoxazole is a sulfonamide monotherapy and that an alternative may be doxycycline and hydroxychloroquine, an alkalinising agent [1,60,61]. However, it is noteworthy to remember that trimethoprim-sulfamethoxazole has replaced cyclines, which had the reputation of not crossing the blood-brain barrier in adequate amounts, an issue that has not yet been confirmed for doxycycline [10]. That is why we first used a combination of doxycycline, hydroxychloroquine and trimethoprim-sulfamethoxazole to manage *T. whipplei *encephalitis. However, recent evidence suggests that sulfadiazine is preferable; its efficacy is comparable to that of the sulfomethoxazole, but a higher dose of sulfadiazine can be used to improve its ability to cross the blood-brain barrier [10]. Antibiotics had a quick effect. However, 3 of our 5 patients presented with clinical relapse despite at least 18 months of therapy. When the treatment was reintroduced, a quick effect was again observed. As for classic Whipple's disease, the optimal treatment for *T. whipplei *encephalitis has not yet been determined [10]. Overall, the data emphasise the need for long-term treatment and lifelong follow-up.

## Conclusion

We describe a clinical entity that presents with unexplained progressive dementia, generally associated with ataxia or recent obesity, and responds dramatically to antibiotics. We suspect that many more cases may be observed, as 3 of the recorded patients were hospitalised in Marseille, where the study was performed.

## Competing interests

The authors declare that they have no competing interests.

## Authors' contributions

DR designed the study and analysed the data. FF, FN, CP, JCA, PC, HL and JP collected and analysed the data. FF and DR wrote the manuscript. All authors read and approved the final manuscript.

## Pre-publication history

The pre-publication history for this paper can be accessed here:

http://www.biomedcentral.com/1471-2334/11/171/prepub

## Supplementary Material

Additional File 1 **Figure S1**. Magnetic resonance spectroscopy results* of patient 1. Click here for file

Additional file 2 **Table S1**. Summary of 20 patients with *T. whipplei *chronic encephalitis Click here for file
