# Supplementary material for: Progressive dementia associated with ataxia or obesity in patients with Tropheryma whipplei encephalitis
Source: BMC Infect Dis. 2011 Jun 15;11:171. doi: 10.1186/1471-2334-11-171 (PMC3141410; doi:10.1186/1471-2334-11-171)
Supplement: Additional File 1 — Figure S1. Magnetic resonance spectroscopy results* of patient 1. [file 1471-2334-11-171-S1.DOC]

**Additional file 2, Figure S1.**

**Title: Magnetic resonance spectroscopy results of patient 1*.***

**Description:** In our patient, magnetic resonance spectroscopy was characterized by a decrease in the Nacetyl aspartate and myo-inositol peaks (neuronal and glial injury), an increase in lipids and/or macromolecules, while lactate and other amino-acids suggestive of abscess were not detected. Finally, the absence of increase of the choline over creatine ratio discarded the hypothesis of a demyelinating or tumoral lesion. These anomalies, although not specific, disappeared after 6 months of treatment as magnetic resonance imaging and the neurologic status of the patient dramatically improved. At the time of the relapse, the same abnormal pattern than those observed at the time of diagnosis was detected. Seven-teen months after the reintroduction of the treatment, improvements are observed but they were worse in comparison to those obtained for the first treatment.

***
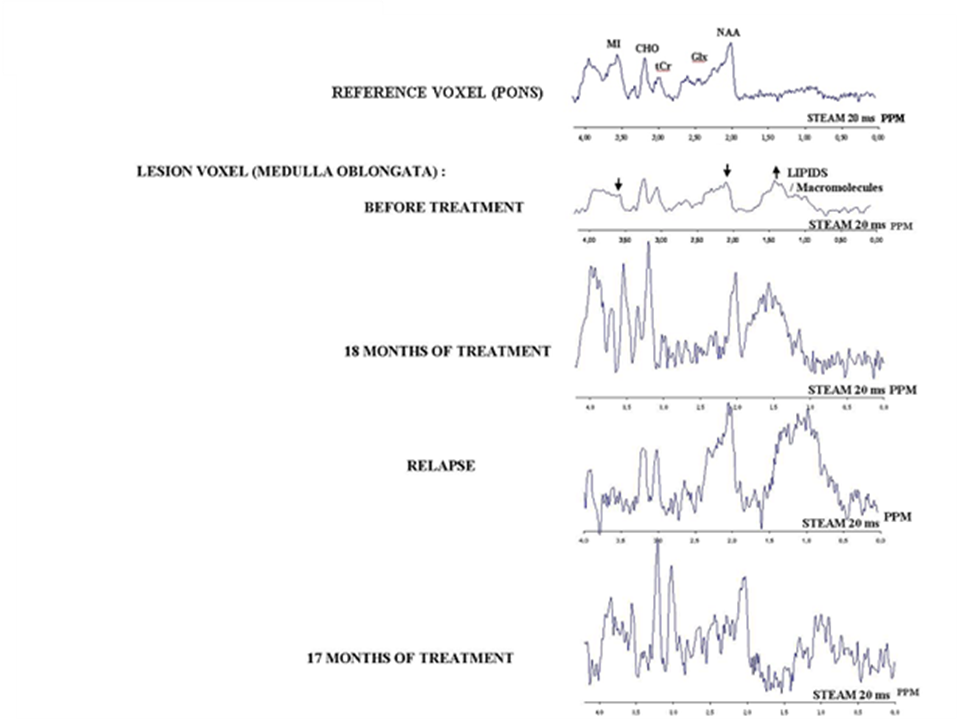
***
