# Supplementary material for: Progressive dementia associated with ataxia or obesity in patients with Tropheryma whipplei encephalitis
Source: BMC Infect Dis. 2011 Jun 15;11:171. doi: 10.1186/1471-2334-11-171 (PMC3141410; doi:10.1186/1471-2334-11-171)
Supplement: Additional file 2 — Table S1. Summary of 20 patients with T. whipplei chronic encephalitis [file 1471-2334-11-171-S2.DOC]

**Additional file 1, Table S1.**

**Title:** Summary of 20 patients with *T. whipplei* chronic encephalitis.

**Description:** The characteristics of our 5 patients with *T. whipplei* encephalitis and 15 patients from the literature are summarised in this Table.

| **Patients**  [Respective references] | **Sex/**  **Age (y)** | **Neurological signs** | **Extraneurological signs** | **Specific diagnosis** | | **Pretreatment examinations** | **Treatment** | | **Follow-up** | |
| --- | --- | --- | --- | --- | --- | --- | --- | --- | --- | --- |
| **CERTAIN DIAGNOSIS OF *T. WHIPPLEI* ENCEPHALITIS** | | | | | | | | | | |
| ***Diagnosis based on T. whipplei PCR of CSF and/or brain biopsy*** | | | | | | | | | | |
| ^1^ Patient 1 | M/39 | **First symptoms**: Dysarthria  **Symptoms:** Dysarthria, cognitive impairment (dementia), choreiform movements, apathy, depression and intermittent diplopia | **Previous:** 3 years before, unexplained diarrhoea for 3 weeks, cytolytic hepatitis, weight gain  **Contemporary:** Dysphagia, cytolytic hepatitis, weight gain | **Brain biopsy:**  Astrocytar gliosis and neoangiogenesis  PAS -, IHC -, regular PCR : -, repeat PCR +  **First CSF:**  Regular PCR: - Repeat PCR: +  **Second CSF:**  Regular PCR: -  Repeat PCR: +  **Gastric and SB biopsy:**  PAS -, IHC -, PCR -  **Saliva:** PCR -  **Stools**: PCR - | | **SGPT** = 123 UI/L and **SGOT** = 51 UI/L.  **CSF**: 4 g/l of protein, glucose level normal, no cells  **Brain CT scan**: Somewhat diminished density involving the right lenticuloinsular area and diminished density of the right frontal convexity  **Brain MRI:** Several high-signal lesions (T2) with a heterogenous contrast (T1) in the cerebral trunk, the anterior hypothalamus, the anterior limbs of the internal capsules and the right centrum semi-ovale. | Doxycycline  200 mg  and  hydroxy-chloroquine  200 mg 3 times/day,  and  TMP-SMX  320-1,600 mg/day 3 times/day  for  18 months | | Quick improvement.  Relapse one year after the end of the treatment.  New treatment for 52 months.  Quick improvement.  No sequelae. | |
| ^1^ Patient 2 | M/35 | **First symptoms:** Dysuria and hypothalamic signs (impotence)  **Symptoms:** Gait ataxia, vertiginous sensation and pyramidal syndrome | **Previous:** Asthenia  **Contemporary:** Asthenia | **First CSF:**  Regular PCR: - Repeat PCR: +  **Second CSF:**  Regular PCR: -  Repeat PCR: +  **Gastric and SB biopsy:**  PAS -, IHC -, PCR -  **Saliva:**  Regular PCR: - Repeat PCR: +  **Stools**:  Regular PCR: - Repeat PCR: + | | **CSF**: 2 g/l of protein, glucose level normal, 9 white blood cells/mm^3^.  **Brain MRI:** High-signal lesions (T2) in the centrum ovale, particularly in the periventricular area on the left; one of the same type of lesion was observed in the left cerebellar area. A lesion that increased after gadolinium injection was present in the left parieto-occipital area in the white matter. | Doxycycline  200 mg  and  hydroxy-chloroquine  200 mg 3 times/day,  and  TMP-SMX  320-1,600 mg/day 3 times/day  for  18 months | | Quick improvement.  No sequelae.  Four years of follow-up. | |
| ^1^ Patient 3 | M/52 | **First symptoms:** Cerebellar form (dysarthria and gait ataxia), cognitive impairment (memory impairment and disorientation), and pyramidal syndrome | **Previous:** Unexplained acute diarrhoea, abdominal pain and fever one month before  **Contemporary:** Dysphagia, abdominal pain, fever | **First CSF:**  Regular PCR: - Repeat PCR: +  **Second CSF:**  Regular PCR: -  Repeat PCR: +  **Gastric and SB biopsy:**  PAS -, IHC -, PCR -  **Saliva:** PCR -  **Stools**: PCR - | | **SGPT** = 107 UI/L and **SGOT** = 51 UI/L  **CSF**: 0.79 g/l of protein, glucose level normal, 9 white blood cells/mm^3^ (80% lymphocytes and 20% mononuclear cells).  **Brain-CT scan:** Normal  **Brain-MRI**: Normal | Doxycycline  200 mg  and  hydroxy-chloroquine  200 mg 3 times/day,  and  TMP-SMX  320-1,600 mg/day 3 times/day  for  18 months | | Quick improvement  No sequelaes Relapse one year after the end of the treatment, with a discrete cerebellar syndrome and a weight gain of 12 kg. New treatment for 12 months.  Quick improvement two months later, with the loss of 7 kg.  No sequelae. | |
| ^1^ Patient 4 | M/53 | **First symptoms:** Gait ataxia, cognitive impairment (memory impairment and confusion), central nystagmus, and pyramidal syndrome | **Previous:** Unexplained acute diarrhoea three weeks before  **Contemporary:** None. | **First CSF:**  Regular PCR: - Repeat PCR: +  **Second CSF:**  Regular PCR: -  Repeat- PCR: +  **Gastric and SB biopsy:**  PAS -, IHC -, PCR –  **Stools**: PCR -  **Blood**: PCR - | | **CSF**: 0.44 g/l of protein, glucose level normal, 20 white blood cells/mm^3^ (mainly lymphocytes).  **Brain-MRI:** Normal | Ceftriaxone  2 g/day  and  TMP-STX  320-1,600 mg/day for 15 months | | Slight improvement.  Few sequelae.  We were not able to continue contact with this patient. | |
| ^1^ Patient 5 | F/33 | **First symptoms:** Headache, diplopia and seizure  **Symptoms:** Cerebellar form (dysarthria and gait ataxia), cognitive impairment (reasoning impairment), choreiform movements, oculomasticatory myorhythmia, right facial paralysis, nystagmus, and muscle weakness | None | **Brain biopsy:** PAS -, IHC -, PCR -  **CSF:** PCR +  **Blood:** PCR –  **Gastric and SB biopsy:** PAS -, IHC -, PCR –  **Saliva:** PCR –  **Stools:** PCR - | | **CSF:** 0.38 g/l of protein, glucose level normal, 22 white blood cells/mm^3^ (mainly lymphocytes).  **Brain MRI:** Mass lesion on the head of left caudate nuclei | Ceftriaxone  2 g/day  and  TMP-STX  320-1,600 mg/day  for 18 months;  Doxycycline  200 mg  and  hydroxy-chloroquine  200 mg 3 times/day  and  sulfadiazine  1g/day 3 times/day | | Slight improvement.  Few sequelae.  Relapse six months after the end of the first treatment.  Slight improvement.  Few sequelae. | |
| ^2^ Patient 6  [30] | F/72 | **First symptoms:**  Personality changes (very anxious), apathy, and cognitive impairment (memory impairment and confusion)  **Symptoms**: Gait ataxia, cognitive impairment (disorientation), extrapyramidal signs, and upper motor neuron disorder | **Previous**: Weight loss  **Contemporary**: Weight loss | **Serum:** PCR +  **CSF:** PCR +  **Duodenum and ileum biopsies:** PAS - | | **CSF**: 45 white blood cells/mm^3^, (mainly lymphocytes) and elevated protein content  **EEG**: Slowing of the background activity and recurrent diffuse synchronous slow and sharp wave discharges.  **Brain MRI**: Mild cortical and subcortical atrophy, with diffusely distributed lacunary lesions that were hypointense on T1-weighted and hyperintense on T2-weighted images. No enhancement after gadolinium administration. | TMP-SMX | | Quick improvement.  No sequelae. | |
| ^2^ Patient 7 [34,37] | M/72 | **First symptoms:** Dizziness  **Symptoms**: Cognitive impairment (memory impairment and incoherent speech), ataxia, supranuclear ophthalmoplegia, and hypersomnia | **Previous**: Anorexia, fatigue  **Contemporary**: Anorexia, fatigue | **CSF:** PCR +  **Duodenal biopsy:**  PAS - | | **Brain MRI:** Significant T1, T2, and fluid-attenuated inversion recovery signalled abnormalities in the cerebellar vermis, bilateral cerebellar peduncles, and superior and posterolateral medulla as well as bilateral foci enhancement in the cerebellar subcortical white matter. | Ceftriaxone and streptomycin, TMP-SMX | | No improvement.  Death. | |
| ^2^ Patient 8  [35] | M/44 | **First symptoms:**  Focal jerks of the left hand and the forearm  **Symptoms:** Cognitive impairment, supranuclear ophthalmoplegia and myoclonus of the left arm and shoulder | None | **CSF:** PCR + from 3 consecutive lumbar punctures  **Duodenal biopsy:**  PAS - | | **CSF:** Pleocytosis (10-36/mm^3^) and mildly increased CSF protein (0.54-1 g/l).  **EEG:** Intermittent bilateral frontotemporal theta activity and no epileptiform discharges.  **Brain MRI:** Bilateral lesions around the parahippocampal gyri, the third ventricle and dorsal pons consistent with limbic encephalitis extending to the upper brainstem. | TMP-SMX and ceftriaxone.  Adjuvant immunotherapy with polyvalent immunoglobulins | | Slow improvement. | |
| ^2^ Patient 9  [36] | M/46 | **First symptoms:**  Cognitive impairment (memory impairment)  **Symptoms:**  Ataxia, cognitive impairment (confusion and memory impairment), supranuclear ophthalmoplegia, seizures, SIADH, and insomnia | None | **CSF:** PCR+  **Duodenal biopsy:**  PAS -, PCR - | | **CSF:** 464 white blood cells/mm^3^ (90% polymorphs, 10% lymphocytes) and decreased CSF protein (0.03 g/l) and glucose (1.6 mmol/l).  **November** **2003** **Brain -MRI:** Atrophy and gliosis of right hippocampal formation.  **March 2004 Brain MRI:**  Development of nodular enhancing lesions (right temporal lobe, caudate nuclei, anterior commissure, right globus pallidus, left insula cortex, left hippocampal formation, lenticular striate territory, mesencephalon and perivascular spaces). | Meropenem for 2 weeks, TMP-SMX for 13 months | | Slow improvement.  Sequelae. | |
| ^2^ Patient 10  [43] | F/39 | **First symptoms:** Speech disturbance and weakness  **Symptoms**:  Cognitive impairment (memory impairment), depression, decreased level of conscientiousness, personality change, seizures, supranuclear ophthalmoplegia, left hemiplegia, urinary incontinence, and paresthesias | **Previous**: Unexplained acute diarrhoea 10 months before, associated with a petechial rash  **Contemporary**: Fever | | **Blood sample:** PCR +  **Brain biopsy:** PAS +, EM + and PCR +  **Digestive biopsy:** PAS - | **Brain MRI**: Bilateral hemispheric high signal on T2-weighted images. | | TMP-SMX | | Slow improvement.  Sequelae. |
| ***Diagnosis based on positive T. whipplei PCR on samples other than CSF and brain biopsy*** | | | | | | | | | | |
| ^2^ Patient 11  [28] | M/65 | **First symptoms**: Gait ataxia, supranuclear ophthalmoplegia, hypothalamic signs (daytime somnolence and nocturnal insomnia)  **Symptoms**: Decreasing level of conscientiousness, hemiparesia, myorhythmia, myoclonus, and upper motor neuron disorder | None | **Blood sample:**  PCR +  **Brain autopsy:**  PAS +  **Digestive biopsy:**  PAS - | | **CSF**: Normal.  **Brain MRI**: Mild to moderate cortical atrophy without focal abnormalities. | Penicillin and streptomycin, followed by TMP-SMX | | Quick  Improvement.  Sudden death due to a mesenteric thrombosis. | |
| ^2^ Patient 12[27] | F/47 | **First symptoms:** Oculomasticatory myorhythmia and nystagmus  **Symptoms**:  Cognitive impairment (memory impairment), cerebellar form (dysarthria and ataxia), hypothalamic signs (insomnia and intermittent hypersomnolence), depression, supranuclear gaze palsy, and upper motor neuron disorder | **Previous**: Arthritis.  **Contemporary:** Fever, submandubular lymph node enlargement | **CSF:** PAS -  **Duodenal biopsy**: PAS -, PCR + | | **CSF**: Protein levels from 0.5 to 0.55 g/l, normal glucose level, and 0 to 70 mononuclear cells.  **EEG**: Mildly generalised slow background.  **Brain-CT**: Normal.  **Brain-MRI**: Arnold-Chiari type 1 malformation with no brainstem compression. | Ceftriaxone 2g/d for 6 months  TMP-SMX | | Improvement  Recurrence with TMP-SMX  Sequelaes | |
| ^2^ Patient 13  [31] | F/54 | **First symptoms:** Blurred vision  **Symptoms**:  Gait ataxia, supranuclear ophthalmoplegia, myorhythmia, extra-pyramidal signs and upper motor neuron disorder | None | **Jejunal biopsy:**  PAS - and PCR + | | **Brain-MRI**: A venous angioma in the left cerebellar hemisphere. | TMP-SMX followed by ceftriaxone | | Slow improvement  No sequelaes | |
| **POSSIBLE DIAGNOSIS OF *T. WHIPPLEI* ENCEPHALITIS** | | | | | | | | | | |
| ***Diagnosis based on positive PAS-staining and electron microscopy of brain biopsy*** | | | | | | | | | | |
| ^2^ Patient 14  [38] | M/40 | **First symptoms:** Headache and seizures  **Symptoms**: Cerebellar form (gait ataxia and dysarthria), cognitive impairment (dementia, memory impairment, confusion), decrease level of conscientiousness, urine incontinence, and upper motor neuron disorder | None | **Brain necropsy :** PAS + and EM +  **Digestive biopsy:** PAS - | | **CSF**: Pleocytosis and occasional level of protein. | -* | | Death | |
| ^2^ Patient 15  [39] | M/32 | **First symptoms:** Headache  **Symptoms:** Decreasing level of conscientiousness, cognitive impairment (dementia, disorientation, memory impairment), apathy, upper motor neuron disorder, and seven nerve palsy | None | | **Brain biopsy:** PAS + and EM +  **Duodenal and jejunal biopsies:**  PAS - | **CSF**: Normal excepted oligoclonal bands.  **Brain** **MRI**: Large high-signal mass lesions with peripheral ring enhancement after gadolinium injection. | | Penicillin and streptomycin for 2 weeks, followed by TMP-SMX for 11 months | | Slow improvement. Sequelae. |
| ^2^ Patient 16 [32] | M/58 | **Symptoms**:  Cerebellar form (dysarthria and gait ataxia), cognitive impairment (memory impairment), supranuclear ophthalmoplegia, dizziness, personality change, nystagmus, upper motor neuron disorder, hypothalamic signs (impotence), and paresthesias | **Previous**: Arthralgias  **Contemporary**: Low-grade fever | **CSF:** PAS -  **Brain biopsy:** PAS + and EM +  **Jejunal biopsy:**  PAS - | | **CSF:** 100 mg/dl of protein, 99 mg/dl of glucose, 9,330 red blood cells and 4 lymphocytes.  **Brain CT scan:** Round areas of low density without mass effect in the right medial temporal lobe and right pontine tegmentum. After the infusion of intravenous contrast material, these lesions demonstrated increased intensity. There was slight central and cortical atrophy. | ChloramphenicolTCN | | Death 6 months after diagnosis | |
| ^2^ Patient 17 [40] | F/63 | **First symptoms:** Myorhythmia  **Symptoms**: Cerebellar form (ataxia and dysarthria), cognitive impairment (memory impairment), decreased level of consciousness, supranuclear ophthalmoplegia, myoclonus, hemiparesia, depression, extra-pyramidal signs, and personality changes | **Previous**: Arthralgia for 10 years  **Contemporary**: None | **Jejunal biopsy:**  PAS -, PCR -  **Brain necropsy:** PAS+ and EM + | | **CSF**: Normal.  **EEG**: Normal.  **Brain CT scan**: Normal.  **Brain MRI**: Normal. | No antibiotic | | Death | |
| ^2^ Patient 18  [41] | F/63 | **First symptoms:** Cognitive impairment (memory impairment), somnolence and ataxia  **Symptoms**: Cognitive impairment (disorientation), supranuclear ophthalmoplegia, hypothalamic signs (hyperphagia), and upper motor neuron disorder | None | **Brain autopsy:**  PAS + and EM +  **Digestive biopsy:**  PAS - | | **CSF**: Normal.  **Brain MRI**: Abnormal high signal intensity in the hypothalamus, uncus, and medial temporal lobes on T2-weighted images. | Penicillin, TMP-SMX, rifampin, chloramphenicol | | Death | |
| ^2^ Patient 19  [42] | F/55 | **First symptoms:** Somnolence, cognitive impairment (confusion, and memory impairment)  **Symptoms**: Cognitive impairment (disorientation), decrease level of conscientiousness, oculomasticatory myorhythmia, and supranuclear ophthalmoplegia | **Previous**: Arthralgia  **Contemporary**: Arthralgia | **Brain autopsy:**  PAS + and EM +  **Duodenal biopsy:** PAS - | | **CSF**: Normal.  **EEG**: Diffusely slow with frontal, intermittent, rhythmic delta waves.  **Brain RMI**: Increased signal density around third ventricle. | None | | Death | |
| ***Diagnosis based exclusively on oculomasticatory myorhythmia that is considered by some authors as pathognomonic of T. whipplei*** | | | | | | | | | | |
| ^2^ Patient 20  [27] | M/54 | **First symptoms:**  Cognitive impairment (dementia), supranuclear ophthalmoplegia, extra pyramidal signs  **Symptoms:** Gait ataxia, cognitive impairment (memory impairment), depression, and oculomasticatory myorhythmia | None | Oculomasticatory myorhythmia  **Digestive biopsy:**  PAS - | | - | TCN 1g/d.  Prior treatment lost to follow-up. | | No benefit | |

^1^: Patients were seen and followed by one of the authors (DR) in consultation.

^2^: Patients were from the literature review.

*: The diagnosis of *T. whipplei* infection was made after post-mortem examination.

SGPT : Serum glutamate pyruvate transaminases

SGOT : Serum glutamic oxaloacetic transaminases

PAS+: Light microscopy showing PAS-positive macrophages in infected tissues

EM+: Electron microscopy showing Whipple bacilli in infected tissues

PCR TW+: Polymerase chain reaction showing *T.* *whipplei* DNA

MRI: Magnetic resonance imaging

TMP-SMX: Trimethoprim-sulfamethoxazole

TCN: Tetracycline

FLAIR: Fluid attenuation inversion recovery

SIADH: Syndrome of inappropriate ADH secretion
